# Supplementary material for: Human Disturbance Increases Health Risks to Golden Snub-Nosed Monkeys and the Transfer Risk of Pathogenic Antibiotic-Resistant Bacteria from Golden Snub-Nosed Monkeys to Humans
Source: Animals (Basel). 2023 Oct 3;13(19):3083. doi: 10.3390/ani13193083 (PMC10572025; doi:10.3390/ani13193083)
Supplement: Supplementary file 1 [file animals-13-03083-s001.zip › animals-2533952-supplementary.pdf]

Table S1. Samples selected by micro-PITA for metagenomic sequencing.

Treatments	Sample							
Wild group (W group)	W36	W42	W10	W40	W15	W41	W34	W38
	W9	W7	W5	W3	W13	W44	W21	
human disturbance group (HD group)	HD1	HD4	HD7	HD31	HD17	HD50	HD41	HD34
	HD16	HD35	HD33	HD40	HD44	HD38	HD47	

Table S2 Characteristics of high quality MAGs

	Bin Id	Contigs Num	N50（contigs）（bp）	Mean contig length（bp）	Completeness（%）	Contamination（%）	Bins converage	Cotigs contain (%)
	bin570	502	2139537	23483	80.05	5.87	37	0.001944531
	bin580	405	3443	3256	57.82	2.78	95	0.001568795
	bin134	96	1524783	100348	62.05	5.8	147	0.000371862
	bin399	455	2823	2677	50.4	0.67	37	0.001762473
	bin379	19	83111	48057	50.83	0	171	7.35978E-05
	bin641	295	2429	2429	51.31	0.81	14	0.001142702
	bin360	363	2860	2794	51.37	0.67	27	0.001406105
	bin671	432	2363	2393	51.46	1.51	24	0.001673381
	bin388	390	3093	2852	53.03	3.52	72	0.001510691
	bin333	52	42622	24508	53.49	3.14	69	0.000201425
	bin248	272	2859	2701	54.23	3.37	28	0.00105361
	bin43	19	115017	65510	54.89	0.24	144	7.35978E-05
	bin400	351	3463	3147	54.96	0.45	37	0.001359622
	bin241	536	5103	4086	57.24	1.75	58	0.002076232
	bin239	341	3888	3472	57.76	0	27	0.001320886
	bin376	101	17424	13126	57.9	0.67	171	0.00039123
	bin27	195	10300	7684	67.01	0	455	0.000755346
	bin32	209	4059	3483	64.39	0	34	0.000809575
	bin410	23	66135	41129	61.6	0	189	8.9092E-05
	bin12	68	17962	11873	75.67	0.13	29	0.000263403
	bin34	48	22304	13578	61.94	0.16	69	0.000185931
	bin2	51	63217	41040	99.33	0.17	77	0.000197552
	bin36	339	5307	4464	74.34	0.17	16	0.001313139
HD	bin504	66	78904	36822	99.19	0.2	60	0.000255655
	bin291	52	48449	33590	69.62	0.27	93	0.000201425
	bin200	95	42686	18884	97.09	0.34	38	0.000367989

bin383	268	4338	3848	71.02	0.4	26	0.001038116
bin431	223	12765	8605	95.3	0.45	58	0.000863805
bin469	124	15063	10105	75.07	0.51	118	0.000480322
bin438	308	8382	6003	94.3	0.56	38	0.001193059
bin6	329	4759	4144	83.5	0.58	24	0.001274403
bin638	292	13836	8393	95.86	0.62	19	0.001131081
bin56	90	36174	22676	97.32	0.67	54	0.000348621
bin394	95	27349	15780	76.44	0.67	70	0.000367989
bin152	225	12626	9040	90.25	0.69	50	0.000871553
bin600	25	108850	83463	91.61	0.72	390	9.68392E-05
bin257	30	150574	67116	80.38	0.75	72	0.000116207
bin5	338	9161	6310	95.97	0.81	43	0.001309266
bin692	31	121699	56853	79.35	0.92	114	0.000120081
bin711	312	6081	4838	84.33	0.94	44	0.001208553
bin23	73	44694	24280	95.39	1.01	69	0.00028277
bin660	142	21997	12675	89.26	1.01	55	0.000550046
bin145	132	23791	13143	94.92	1.08	30	0.000511311
bin472	209	16859	10971	72.09	1.08	97	0.000809575
bin589	194	18139	11090	94.8	1.34	55	0.000751472
bin273	54	45885	20729	80.85	1.4	46	0.000209173
bin654	252	15850	10149	95.5	1.45	31	0.000976139
bin30	374	3014	2877	60.28	1.46	20	0.001448714
bin693	726	6985	5024	94.72	1.54	36	0.002812209
bin474	129	33283	15394	95.57	1.58	45	0.00049969
bin644	175	21731	12692	88.11	1.6	43	0.000677874
bin497	355	5843	4709	90.88	1.61	31	0.001375116
bin332	74	60012	27326	97.89	1.64	108	0.000286644
bin446	116	36484	23295	96.98	1.67	27	0.000449334
bin378	605	4309	3795	80.13	1.69	61	0.002343508
bin52	261	22973	14965	98.58	1.77	46	0.001011001
bin18	64	88264	33098	95.85	1.89	32	0.000247908
bin515	422	3370	3113	67.79	1.89	45	0.001634645
bin11	184	15064	10943	84.79	1.92	55	0.000712736
bin310	171	8088	6415	64.24	1.95	263	0.00066238
bin485	281	14925	9336	95.97	1.96	56	0.001088472
bin361	87	81682	38466	98.66	2.01	96	0.000337
bin734	282	8412	6411	92.37	2.01	24	0.001092346
bin53	361	8648	6659	91.38	2.01	37	0.001398358
bin284	431	4266	3512	61.13	2.05	31	0.001669507
bin724	418	4719	4156	88.04	2.13	29	0.001619151
bin553	102	22618	12642	70.74	2.13	276	0.000395104
bin403	394	4421	3774	70.68	2.2	94	0.001526185
bin162	58	128189	50018	98.31	2.22	265	0.000224667
bin389	74	23454	14006	89.33	2.25	209	0.000286644

bin46	217	8592	6176	85.86	2.25	56	0.000840564
bin454	110	12322	8243	68.73	2.25	46	0.000426092
bin717	80	103091	43318	99.26	2.31	29	0.000309885
bin511	170	15155	8146	88.24	2.31	44	0.000658506
bin313	147	18324	9418	73.25	2.32	62	0.000569414
bin304	362	3958	3544	67.82	2.32	26	0.001402231
bin395	142	30816	18014	96.06	2.35	37	0.000550046
bin202	350	5173	4154	77.96	2.38	37	0.001355748
bin85	406	6515	5018	91.37	2.48	43	0.001572668
bin442	190	19663	10943	90.96	2.49	71	0.000735978
bin533	155	39478	22114	96.04	2.68	61	0.000600403
bin151	280	13131	8699	94.5	2.68	43	0.001084599
bin28	258	4624	4054	73.65	2.69	15	0.00099938
bin614	375	2631	2649	64.3	2.8	28	0.001452588
bin211	372	4685	3990	81.68	2.84	81	0.001440967
bin13	39	80971	56119	95.04	2.86	52	0.000151069
bin168	549	8919	6158	94.65	2.97	32	0.002126588
bin221	677	7862	5629	94.97	3.02	14	0.002622405
bin131	96	45440	25025	96.67	3.15	96	0.000371862
bin579	98	39077	19702	70.07	3.23	134	0.00037961
bin144	507	5288	4272	84.89	3.25	25	0.001963898
bin216	166	22264	13129	91.82	3.49	68	0.000643012
bin490	427	4801	4089	84.01	3.52	45	0.001654013
bin214	287	6681	5393	77.23	3.52	214	0.001111714
bin73	504	10462	7038	90.72	3.54	24	0.001952278
bin702	76	17417	12588	71.35	3.56	320	0.000294391
bin491	222	20152	13237	93.06	3.58	88	0.000859932
bin687	306	14569	9998	92.86	3.76	26	0.001185311
bin205	461	4909	4206	86.76	3.79	17	0.001785714
bin730	540	4234	3645	80.71	3.84	75	0.002091726
bin645	295	12374	8123	95.72	3.9	97	0.001142702
bin323	365	6044	4987	83.33	3.92	20	0.001413852
bin358	150	13513	8223	92.7	3.93	92	0.000581035
bin551	100	22466	12500	89.89	3.93	44	0.000387357
bin188	258	6711	5005	86.6	3.93	56	0.00099938
bin316	315	11330	7320	95.22	4.03	21	0.001220174
bin300	403	6512	5175	89.75	4.26	24	0.001561047
bin527	71	55445	22225	78.68	4.3	68	0.000275023
bin147	223	12461	7855	93.86	4.31	40	0.000863805
bin175	141	25115	14560	94.63	4.36	84	0.000546173
bin568	166	11173	7611	76.63	4.38	99	0.000643012
bin548	210	18372	12594	93.82	4.47	52	0.000813449
bin7	268	13137	8303	97.93	4.63	39	0.001038116
bin329	250	15837	9002	93.58	4.66	105	0.000968392

	bin566	311	3128	2883	61.1	4.73	111	0.001204679
	bin623	449	4402	3842	77.98	4.81	25	0.001739231
	bin220	313	8170	6564	92.35	4.98	41	0.001212426
	bin126	317	2760	2657	50.85	2.07	24	0.00103002
	bin8	58	27680	17093	52.64	2.45	127	0.000188458
	bin314	57	70651	16175	58.24	2.25	72	0.000185209
	bin414	124	14466	8773	58.32	1.68	119	0.00040291
	bin270	462	3593	3273	59.48	1.72	98	0.001501165
	bin348	98	38187	22150	97.09	0	62	0.000318429
	bin539	102	32505	19309	94.65	0	44	0.000331426
	bin280	177	13058	8570	81.9	0	42	0.000575122
	bin467	71	13341	10355	63.33	0	119	0.000230698
	bin386	341	6521	5342	82.67	0.06	26	0.001108003
	bin524	80	34088	22195	84.17	0.48	51	0.000259942
	bin252	251	15769	8295	77.48	0.66	24	0.000815568
	bin239	278	13664	8219	93.04	0.67	20	0.000903298
	bin271	96	30273	17016	90.46	0.79	72	0.00031193
	bin119	183	12913	8151	94.72	0.81	45	0.000594617
	bin213	115	72691	24360	97.34	0.9	58	0.000373667
	bin337	340	3437	3190	65.8	1.01	32	0.001104753
	bin202	107	13088	9147	82.5	1.12	63	0.000347672
	bin241	57	13035	9984	70.22	1.12	292	0.000185209
	bin569	122	10198	7668	62.84	1.28	216	0.000396412
	bin62	582	2758	2678	63.07	1.29	12	0.001891078
	bin257	339	4323	3822	77.25	1.34	14	0.001101504
	bin248	387	3666	3442	69.16	1.34	28	0.001257469
W	bin373	122	17863	11090	62.65	1.68	297	0.000396412
	bin509	53	40878	22875	92.42	1.69	39	0.000172212
	bin50	449	3228	2992	62.93	1.72	25	0.001458924
	bin580	264	6646	4939	77.87	1.8	112	0.000857808
	bin455	127	21860	10680	68.64	2.01	72	0.000412658
	bin120	52	40550	23225	72.75	2.08	72	0.000168962
	bin559	515	3564	3280	64.85	2.09	25	0.001673376
	bin198	230	7595	5854	85.77	2.24	23	0.000747333
	bin38	311	4656	4209	71.47	2.35	35	0.001010524
	bin197	524	4385	3837	78.43	2.42	61	0.00170262
	bin351	259	11797	8029	92.84	2.49	16	0.000841562
	bin110	182	48727	26338	98.28	2.51	26	0.000591368
	bin80	333	11850	7302	94.38	2.6	27	0.001082008
	bin3	60	56929	29769	98.67	2.67	33	0.000194956
	bin132	174	7190	5244	82.21	2.81	52	0.000565374
	bin183	229	27826	12575	89.52	2.9	117	0.000744084
	bin106	429	5674	4344	86.87	3.12	36	0.001393939
	bin282	126	51091	18995	100	3.15	111	0.000409409

bin577	586	3786	3479	78.53	3.31	30	0.001904075
bin44	459	4827	4146	79.42	3.35	33	0.001491417
bin39	114	47404	18747	96.42	3.36	170	0.000370417
bin141	246	10310	6865	93.79	3.38	46	0.000799322
bin61	361	5583	4672	76.37	3.47	49	0.001172988
bin533	139	14691	8550	68.89	3.58	98	0.000451649
bin188	134	13857	8413	68.5	3.84	74	0.000435403
bin193	153	12506	8003	80.9	3.93	58	0.000497139
bin176	251	16865	8361	95.97	4.26	94	0.000815568
bin553	519	5091	4289	88.55	4.47	31	0.001686374
bin171	407	4120	3665	64.05	4.63	33	0.001322455
bin143	467	3011	2845	72.86	4.64	32	0.001517411
bin375	175	8262	6450	61.88	5	349	0.000568623

Table S3 Rotating component matrix in factor analysis (KMO and Bartlett tests, *P*=0.000002)

	**ARGs or VFs**	**F_1**	**F_2**	**F_3**	**F_4**	**F_5**	**F_6**
	Adherence	-0.138	0.052	-0.171	0.065	-0.854	0.086
	Exoenzyme	-0.03	-0.306	-0.554	0.531	0.229	0.063
	Immune modulation	0.56	-0.175	-0.365	0.061	-0.56	0.09
	Nutritional/Metabolic factor	0.957	0.017	0.128	-0.102	0.074	0.057
Antibiotic resistance genes	Regulation	0.90	-0.046	0.216	-0.214	0.148	0.071
(ARGs)	Stress survival	0.879	0.291	-0.188	-0.021	-0.127	-0.078
	Biofilm	0.231	-0.178	0.651	-0.178	0.447	0.252
	Effector delivery system	0.31	0.694	0.278	0.019	0.075	0.127
	Exotoxin	0.159	0.038	0.745	0.049	0.246	-0.063
	Motility	-0.226	0.691	0.056	0.085	-0.217	0.086
	Others	0.805	0.088	0.355	0.128	0.162	0.078
	Multdrug	-0.194	-0.511	0.02	0.74	-0.041	0.064
Virulence genes (VFs)	Glycopeptide antibiotic	0.263	0.761	-0.412	-0.191	0.18	-0.025
	Lincosamide antibiotic	-0.007	-0.303	0.017	-0.754	0.159	0.153
	Aminoglycoside antibiotic	-0.072	-0.124	0	0.063	0.086	-0.942
Variance Contributions (%)		25.457	14.4085	12.828	10.413	10.296	6.678

Note: The F_1, F_2, F_3, F_4, F_5 and F_6 are the factor loading of factor 1, factor 2, factor 3, factor 4, factor 5 and factor 6. *P*=0.000002 indicates that the factor analysis can be used to analyse the contribution of KEGG pathways in antimicrobial drug resistance and bacterial infectious disease to HR of golden snub-nosed monkeys.
